# Obesity or Overweight, a Chronic Inflammatory Status in Male Reproductive System, Leads to Mice and Human Subfertility

**DOI:** 10.3389/fphys.2017.01117

**Published:** 2018-01-04

**Authors:** Weimin Fan, Yali Xu, Yue Liu, Zhengqing Zhang, Liming Lu, Zhide Ding

**Affiliations:** ^1^Shanghai Key Laboratory for Reproductive Medicine, Department of Histology Embryology, Genetics and Developmental Biology, School of Medicine, Shanghai Jiao Tong University, Shanghai, China; ^2^Reproductive Medicine Center, Shanghai Ruijin Hospital, School of Medicine, Shanghai Jiao Tong University, Shanghai, China; ^3^The Laboratory of Clinical Medicine, Shanghai No.9 People's Hospital, School of Medicine, Shanghai Jiao Tong University, Shanghai, China; ^4^Laboratory of Immune Regulation, Shanghai Institute of Immunology, Shanghai Jiao Tong University School of Medicine, Shanghai, China

**Keywords:** inflammation, reproductive system, overweight and obesity, male, subfertility

## Abstract

Obesity is frequently accompanied with chronic inflammation over the whole body and is always associated with symptoms that include those arising from metabolic and vascular alterations. On the other hand, the chronic inflammatory status in the male genital tract may directly impair spermatogenesis and is even associated with male subfertility. However, it is still unclear if the chronic inflammation induced by obesity damages spermatogenesis in the male genital tract. To address this question, we used a high fat diet (HFD) induced obese mouse model and recruited obese patients from the clinic. We detected increased levels of tumor necrosis factor (TNF-α), interleukin-6 (IL-6), and NOD-like receptor family pyrin domain containing-3 (NLRP3) in genital tract tissues including testis, epididymis, seminal vesicle, prostate, and serum from obese mice. Meanwhile, the levels of immunoglobulin G (IgG) and corticosterone were significantly higher than those in the control group in serum. Moreover, signal factors regulated by TNF-α, i.e., p38, nuclear factor-κB (NF-κB), Jun N-terminal kinase (JNK), extracellular signal-regulated kinase (ERK), and their phosphorylated status, and inflammasome protein NLRP3 were expressed at higher levels in the testis. For overweight and obese male patients, the increased levels of TNF-α and IL-6 were also observed in their seminal plasma. Furthermore, there was a positive correlation between the TNF-α and IL-6 levels and BMI whereas they were inversely correlated with the sperm concentration and motility. In conclusion, impairment of male fertility may stem from a chronic inflammatory status in the male genital tract of obese individuals.

## Introduction

Obesity is a global health problem and the prevalence of obesity has risen substantially in the past three decades. For instance, the number of overweight and obese individuals increased from 857 million in 1980, to 2.1 billion in 2013. Worldwide, the proportion of men who were overweight increased from 28.8% in 1980, to 36.9% in 2013 (Ng et al., 2014).

Generally, obesity is a metabolic disease resulting from behavior and heritable causes. Meanwhile, it is also associated with a number of chronic states including metabolic syndrome, hyperlipidemia, type-2 diabetes, cancer, cardiovascular disease, and infertility (An et al., 2017). Besides these associations between obesity and disease, studies over the past years indicate there are important signaling pathways connecting this metabolic syndrome with the immune system (Tilg and Moschen, 2006). These interactions between metabolism and immune system seem to be orchestrated by several mediators derived from immune cells, adipocytes, and systemic inflammation that are induced by obesity (Wellen and Hotamisligil, 2005). One of the sources of these mediators may be dysfunctional adipocytes (Esser et al., 2014).

There is substantive evidence supporting the notion that obesity is correlated with a chronic inflammatory response based on the identification of abnormal cytokines such as TNF-α and IL-6, along with the activation of pro-inflammatory signaling pathways (Wellen and Hotamisligil, 2005). Many studies found that adipocytes can directly express TNF-α in rodents. This finding formed the basis for suggesting that adipocytes have crucial role in inducing chronic inflammatory responses in obesity. Interestingly, these findings were consistent with a human study showing increased TNF-α production in the adipose tissue of obese individuals, which declined TNF-α production after weight loss (Kern et al., 1995). Moreover, it has been reported recently that the NOD-like receptor family pyrin domain containing-3 (NLRP3), one of the inflammasomes, is associated with obesity and contributes to obesity-induced inflammation (Vandanmagsar et al., 2011).

On the other hand, there is much evidence showing that obesity reduces sperm quality and then impairs male fertility (Teerds et al., 2011; Hammiche et al., 2012; Liu and Ding, 2017), whereas metabolic syndrome, hyperlipidemia, and a pro-inflammatory state are independently linked with male subfertility (Klöting and Blüher, 2014). However, it is unclear how obesity elicits its effects on sperm morphology or function. Namely, it needs to be clarified if it occurs as a consequence of increases in obesity and/or is an indirect associated complication of this condition.

We report here on the use of a Male obese mouse model which was successfully induced by being fed a high fat diet (HFD). In parallel, 272 semen samples from healthy, overweight and obese human males were collected and then analyzed. This endeavor had two goals: (1) To determine if obesity can either induce inflammatory responses or increase the expression of pro-inflammation cytokines in the male genital tract and serum; (2) To determine if the proinflammatory cytokines had effects on relevant signaling pathways mediating control of testicular spermatogenesis or on sperm function in the caudal epididymis.

## Materials and methods

### Animals and obese model establishment

Animal experiments were conducted according to the International Guiding Principles for Biomedical Research Involving Animals, as promulgated by the Society for the Study of Reproduction. This research program was approved by the Ethics Committee of Shanghai Jiao Tong University School of Medicine (NO. A2015-034). C57BL/6 mice (Male: aged 3 weeks) were purchased from Shanghai Laboratory Animal Center, and acclimated in the Animal Center of Jiao Tong University Medical School at least for 1 week prior to experimentation. Male mice were divided in two groups every time: 10 mice were continuously fed a high-fat diet (HFD) containing 23.3% casein, 0.3% L-cysteine, 8.5% corn starch, 11.7% maltodextrin, 20.1% sucrose, 5.8% cellulose, 2.9% soybean oil, 20.7% lard, 5.2% mineral mix, 1.2% vitamin mix, and 0.3% choline bitartrate, and 10 control mice fed a normal diet including 19% casein, 0.2% L-cysteine, 29.9% corn starch, 3.3% maltodextrin, 33.2% sucrose, 4.7% cellulose, 2.4% soybean oil, 1.9% lard, 4.3% mineral mix, 0.9% vitamin mix, and 0.2% choline bitartrate in subsequent 10 weeks. Body weight of all animals and food intake were measured every week.

### Mice serum lipids and apolipoprotein (apo)

The mice serum lipids, such as cholesterol (CHOL), triglycerides (TGL), high density lipoprotein (HDL), and low density lipoprotein (LDL), as well as apolipoprotein including ApoB and ApoE were measured at 14 weeks. Precise levels of these lipids were detected using a Roche COBAS c 311 auto biochemistry analyzer (Roche Diagnostics, Mannheim, Germany).

### Histological analysis

Tissues from both HFD mice and control diet (CD) mice were taken for histological evaluation. Mice testes and a small piece of liver were fixed in Bouin's solution for 24 h, and then stored in 70% ethanol for 2 h. Tissues were embedded in paraffin, then sliced into 5 μm thick sections and mounted on glass slides, followed by dewaxing and rehydration. The specimens were then stained with hematoxylin and eosin (H&E), finally observed using a microscope (Nikon, ECLIPSE E600, Japan).

### mRNA quantification by real-time qRT-PCR

Mice testes, epididymal caput and cauda, prostate, and seminal vesicle were homogenized in the TRIzol reagent (Invitrogen, US). cDNA was prepared from 1 μg RNA using PrimeScript RT Master Mix (TaKaRa, Japan). SYBR green-based quantification real-time PCR was applied to measure IL-6, TNF-α, and NLRP3 expression in testes, epididymal caput and cauda, prostates, and seminal vesicles from both HFD mice and CD mice. The sequences of the primers were as follows: IL-6, forward 5′-TTCTTGGGACTGATGCTGGT-3′, reverse 5′-CCTCCGACTTGTGAAGTGGT-3′; TNF-α, forward 5′-ACGGCATGGATCTCAAAGAC-3′, reverse 5′-GTGGGTG-AGGAGCACGTAGT-3′; NLRP3, forward 5′-CATCAATGCTGCTTCGACAT-3′, reverse 5′-TCAGTCCCACACACAGCAAT-3′. Real-Time PCR was performed on an ABI 7500 (Applied Biosystems, US) using SYBR Premix Ex Taq II (TaKaRa, Japan) according to the manufacturer's protocol. The expression levels of genes were normalized against β-actin and a segment of β-actin was amplified with the forward primer 5′-GGGAATGGGTCAGAAGGACT-3′ and reverse primer 5′-CTTCTCCATGTCG-TCCCAGT-3′.

### Mice serum analyses by ELISA

Testosterone, estradiol, progesterone, corticosterone, IgG, IgM, TNF-α, and IL-6 levels in mice sera were measured at the age of 14 weeks. Testosterone (R&D Systems, USA), estradiol (Cayman Chemical, USA), progesterone (Cayman Chemical, USA), corticosterone (ALPCO, USA), IgG (ICL, USA), IgM (ICL, USA), TNF-α (Anogen, Canada), IL-6 (Anogen, Canada) in sera were detected, respectively, by using the immunoassay kits according to the manufacturer's protocol.

### Protein sample preparation from testes

Mice testes were homogenized in RIPA lysis buffer (Thermo Fisher Scientific, USA) containing protease inhibitor (Thermo Fisher Scientific, USA) on ice for 30 min. Then the tissue lysates were centrifuged at 12,000 × g, 10 min, 4°C. The testicular proteins in the supernatant were collected and the concentrations were determined by the BCA Protein Assay Kit (Thermo Fisher Scientific, USA).

### Western blot analysis

Protein samples (20 μg) were separated by using 12% denaturing polyacrylamide gels, then transferred to polyvinylidene difluoride (PVDF) membranes (Millipore, Germany) using a semi-dry transfer apparatus (Bio-Rad, Hercules, CA, USA). Membranes were blocked with 5% bovine serum albumin (BSA) for 1 h at room temperature and immunoblotting was performed overnight at 4°C with the antibodies: p38 (Cell Signaling Technology, 1:1000), p-p38 (phospho T180 + Y182, Abcam, 1:1000), NF-κB (p65, Cell Signaling Technology, 1:1000), p-NF-κB p65 (phospho S536, Abcam, 1:1000), JNK (SAPK/JNK, Cell Signaling Technology, 1:1000), p-JNK (phospho T183 + Y185, Cell Signaling Technology, 1:1000), ERK1/2 (p44/42, Cell Signaling Technology, 1:1000), p-ERK (p-p44/42, phospho T202 + Y204, Cell Signaling Technology, 1:1000), and NLRP3 (Abcam, 1:2000), followed by incubation with secondary antibody conjugated to HRP (Abgent, San Diego, CA, USA, 1:10000 dilution). Meanwhile, β-actin (Cell Signaling Technology, 1:1000) was used to validate protein loading equivalence simultaneously. Signals were generated by enhanced chemiluminescence (Millipore, Germany) according to the manufacturer's protocol and detected by a Luminescent Image Analyzer (Image Quant LAS 4000, GE imagination at work, USA).

### Semen specimens

Human semen specimens were obtained from Reproductive Medicine Center, Ruijin Hospital, Shanghai Jiao Tong University, School of Medicine. Use of the semen samples was approved by the Ethics Committee of this institution and all experiments were performed in accordance with relevant guidelines and regulations.

All semen specimens, both from normal and obese or overweight donors (20 to 35 years old), were collected and the donors gave written informed consent for the use of their leftover semen samples when all IVF treatments finished. Notably, individuals having a history of long-term medication, varicocoele, and infection as indicated by a large number of leukocytes in the semen were excluded from the study. Furthermore, samples that were hyperviscous and necrozoospermic (sperm viability <70%) were also excluded from the study. A computer assisted semen analyzer (CASA, Hamilton-Thorn Research, Beverly, MA, USA) then evaluated semen specimen quality based on parameters described in the World Health Organization guidelines (WHO, 2010).

### Semen samples preparation and seminal plasma ELISA

Fresh human semen specimens were centrifuged (800 g, 10 min, 4°C) and the supernatant containing seminal plasma proteins were stored immediately at −80°C until further use. TNF-α, IL-6 in seminal plasma were detected using the related immunoassay kits (Anogen, Canada) according to the manufacturer's protocol.

### Statistical analysis

All data were analyzed using SAS 8.2 software, and results are presented as mean ± SD. Comparisons between two groups were made using student's *t*-test appropriately. One-way analysis of variance (ANOVA) test was used assuming a two-tail hypothesis with *P* < 0.05. Differences were considered statistically different when *P* < 0.05.

## Results

### Food intake, body weight, and hepatic morphology

**Figure 1:**
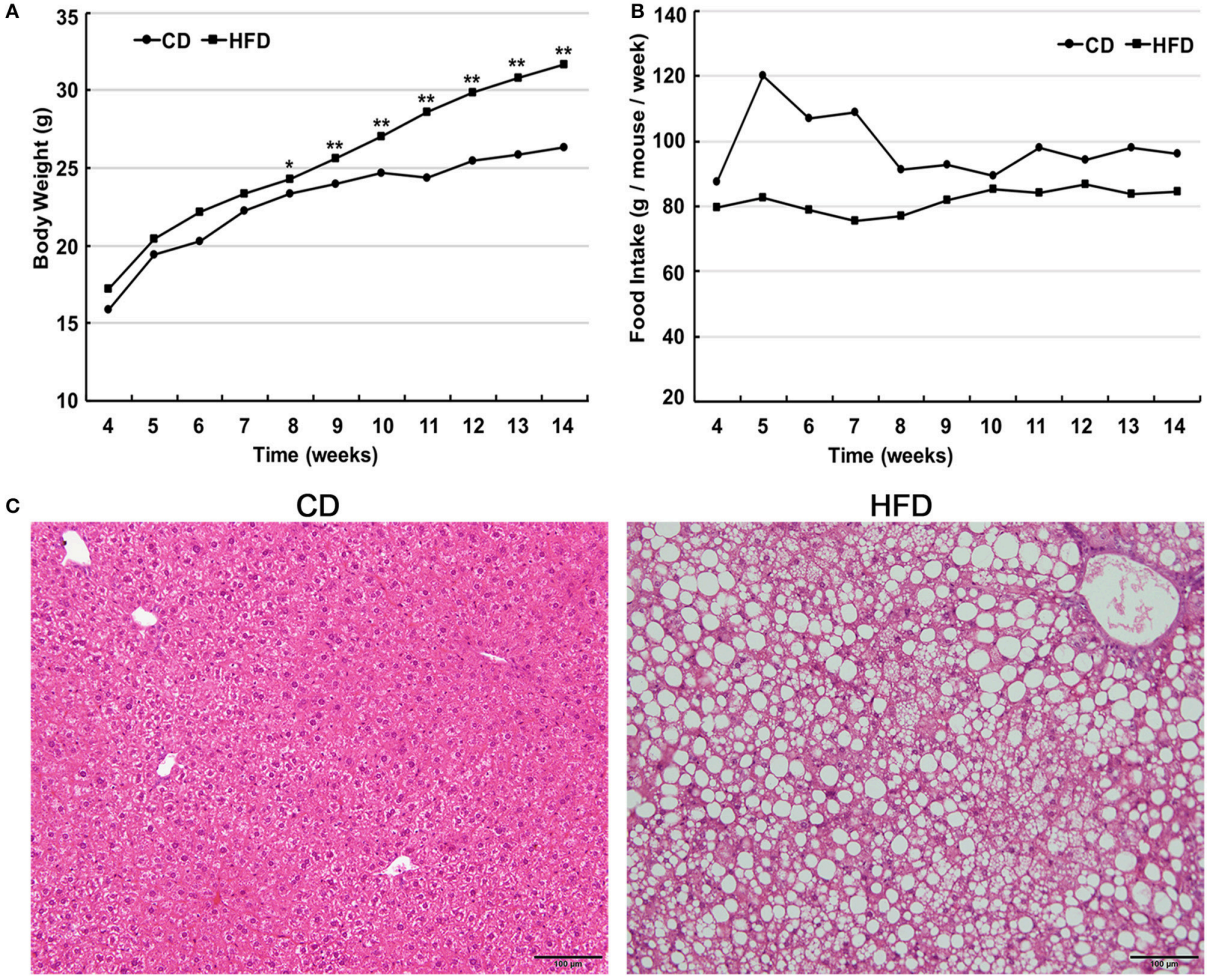
High fat diet feeding establishes obese mouse model. **(A)** Comparison of time-dependent increases in body weight between CD (*n* = 30) and HFD groups (*n* = 30), showed that the body weight gain of the HFD group was significantly greater than that of the CD group (^*^P < 0.05, ^**^P < 0.01). **(B)** Weekly food intake on control diet (CD) and high-fat diet (HFD). **(C)** Hematoxylin and eosin-stained hepatic sections from mice fed either CD or HFD showed a serious hepatic steatosis in HFD fed mice. Scale bar = 100 μm.

Male C57BL/6 mice consumed a high-fat diet for 10 weeks and gained significantly more weight in comparison to that of age-matched littermates fed a normal diet (31.63 ± 1.96 g vs. 26.29 ± 1.05 g, *n* = 30, *P* < 0.05). The differences in body weight between these two groups persisted for 6 weeks (Figure 1A). The food intake by the HFD group per week was always less than that of normal diet group (Figure 1B), but the high-fat diet contains much more calories. Besides, liver morphological analysis clearly indicated that the high-fat diet fed mice had a serious hepatic steatosis and fat vacuoles were evident in almost every hepatic cell (Figure 1C). Then, mice fed a high-fat diet for 10 weeks were placed in the obese group whereas those fed a normal diet were assigned to the control group.

### Serum lipid and hormone profiles

**Figure 2:**
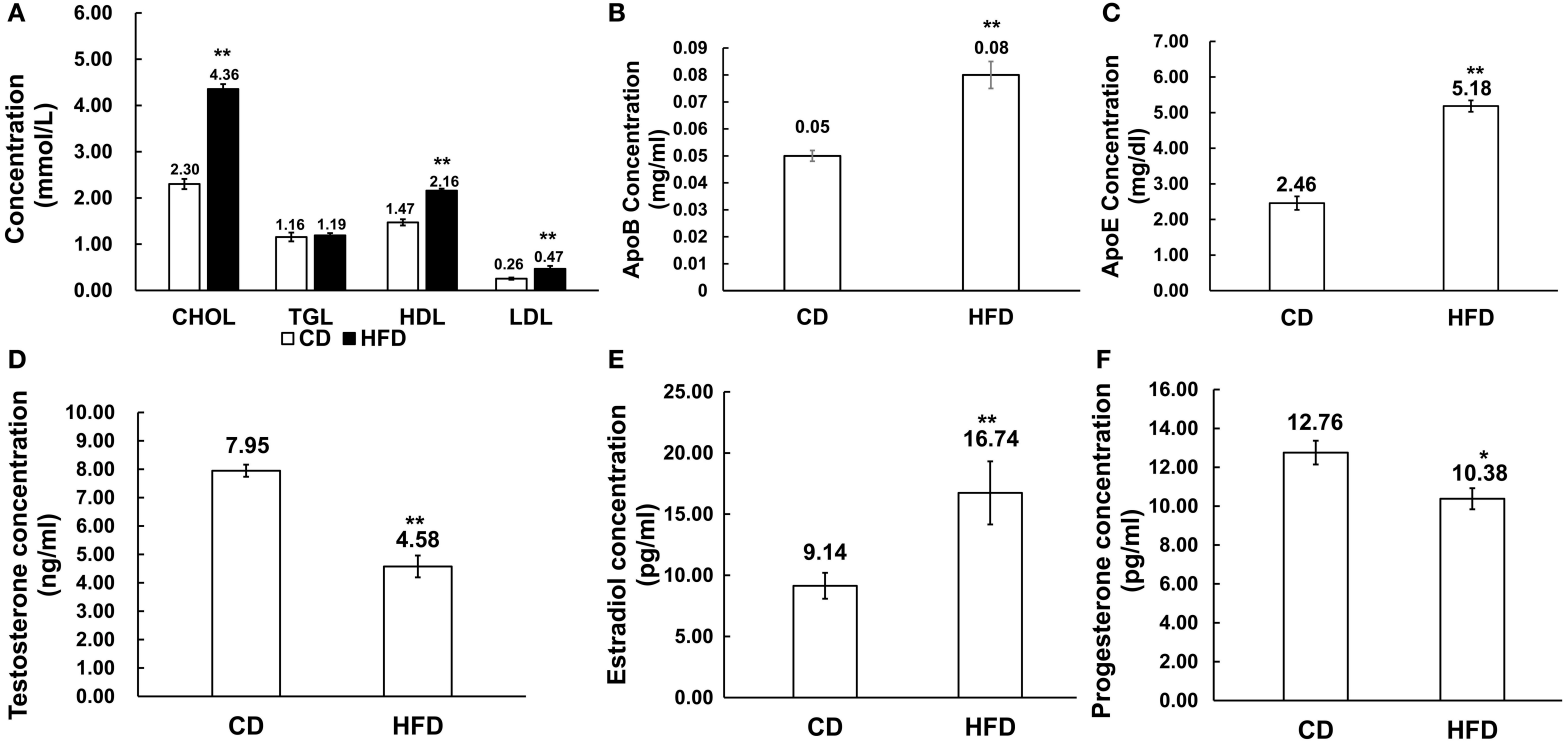
Alteration of serum lipid and sex hormone level profiles in HFD mice. **(A)** Comparison of serum lipid level profiles in CD and HFD groups; CHOL: total cholesterol, TGL, triglycerides; HDL, high density lipoprotein; LDL, low density lipoprotein. **(B,C)** Comparison of ApoB and ApoE profiles in CD and HFD mice. **(D–F)** Comparison of serum sex hormone profiles between CD and HFD groups, including testosterone, estradiol and progesterone. ^*^P < 0.05, ^**^P < 0.01.

Male mice fed with HFD had higher levels of cholesterol (CHOL) (4.36 ± 0.47 mmol/L vs. 2.30 ± 0.65 mmol/L, *n* = 10, *P* < 0.01), HDL (2.16 ± 0.30 mmol/L vs. 1.47 ± 0.25 mmol/L, *n* = 10, *P* < 0.01) and LDL (0.47 ± 0.11 mmol/L vs. 0.26 ± 0.11 mmol/L, *n* = 10, *P* < 0.01) than those in the control group, but there was no difference in their triglyceride (TGL) concentration (1.19 ± 0.40 mmol/L vs. 1.16 ± 0.28 mmol/L, *n* = 10, *P* > 0.05) between the two groups (Figure 2A). Besides, in obese mice, ApoB and ApoE levels were significantly elevated compared to those in control mice (0.08 ± 0.03 g/L vs. 0.05 ± 0.007 g/L, 5.18 ± 0.86 mg/dl vs. 2.46 ± 1.00 mg/dl, *n* = 10, *P* < 0.01; Figures 2B,C).

Serum estradiol level was much higher in obese mice than that in the control group (16.74 ± 1.06 pg/ml vs. 9.14 ± 2.58 pg/ml, *n* = 10, *P* < 0.01), and inversely, serum testosterone (4.58 ± 1.44 ng/ml vs. 7.95 ± 0.80 ng/ml, *n* = 10, *P* < 0.01) and progesterone (10.38 ± 1.43 pg/ml vs. 12.76 ± 1.62 pg/ml, *n* = 10, *P* < 0.05) levels significantly decreased in obese mice compared to those in the control group (Figures 2D–F).

### Effects of high-fat diet on testicular morphological structure

**Figure 3:**
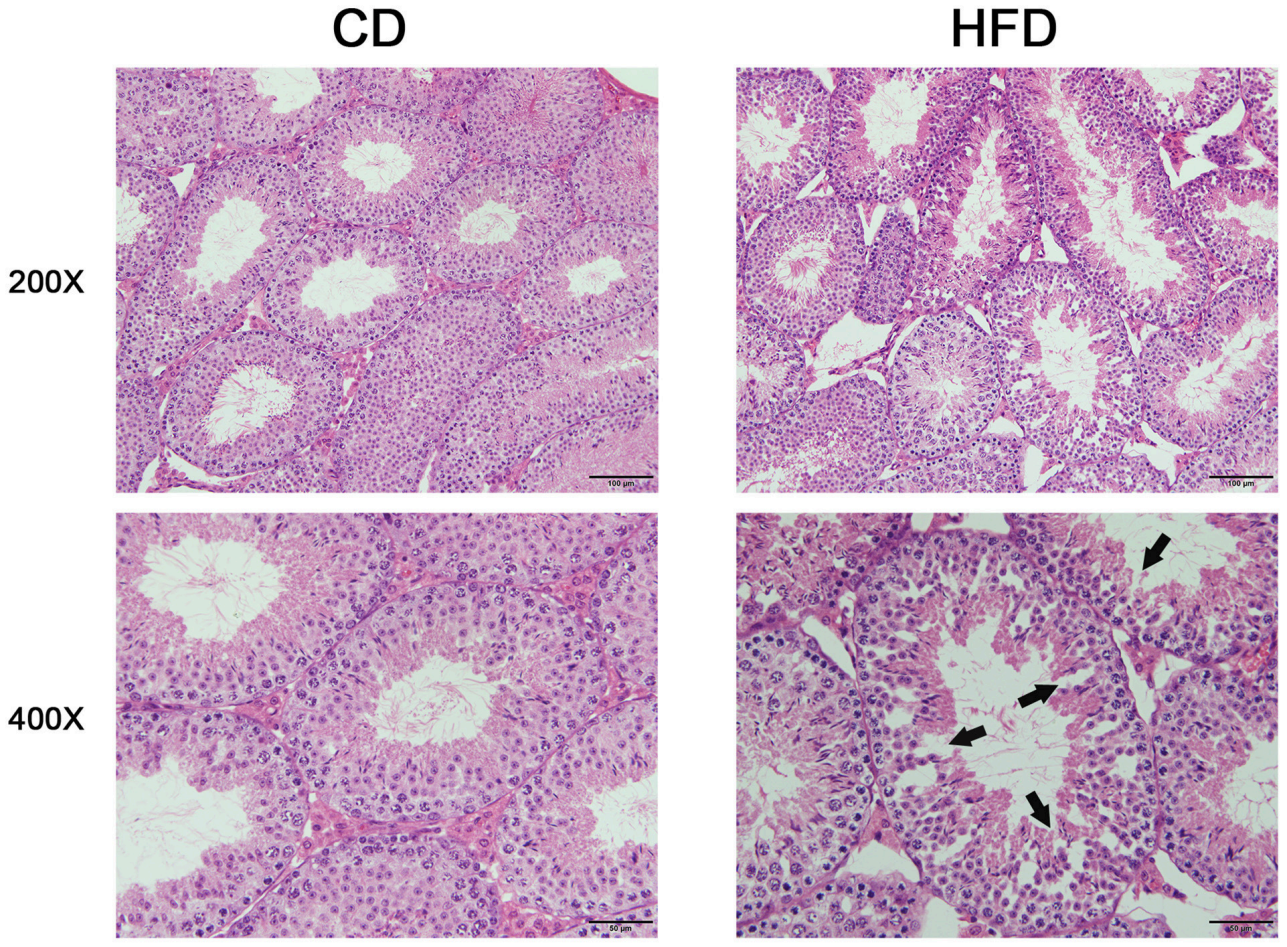
Comparison of testicular morphology in CD and HFD mice. Hematoxylin and eosin-stained testicular sections, both imaged with 20× and 40× objectives, revealed loosely arranged spermatogenic cells (arrows) on the seminiferous epithelium in HFD mice. Scale bar = 50 or 100 μm.

Morphological analysis of the testes indicated that obese mice had an abnormal testicular structure compared with that of normal mice (Figure 3). The seminiferous epithelia were atrophied in high-fat diet mice and cell adhesion between spermatogenic cells and Sertoli cells were impaired and loosely arranged.

### Levels of pro-inflammatory cytokines, corticosterone, IgG, and IgM in sera

**Figure 4:**
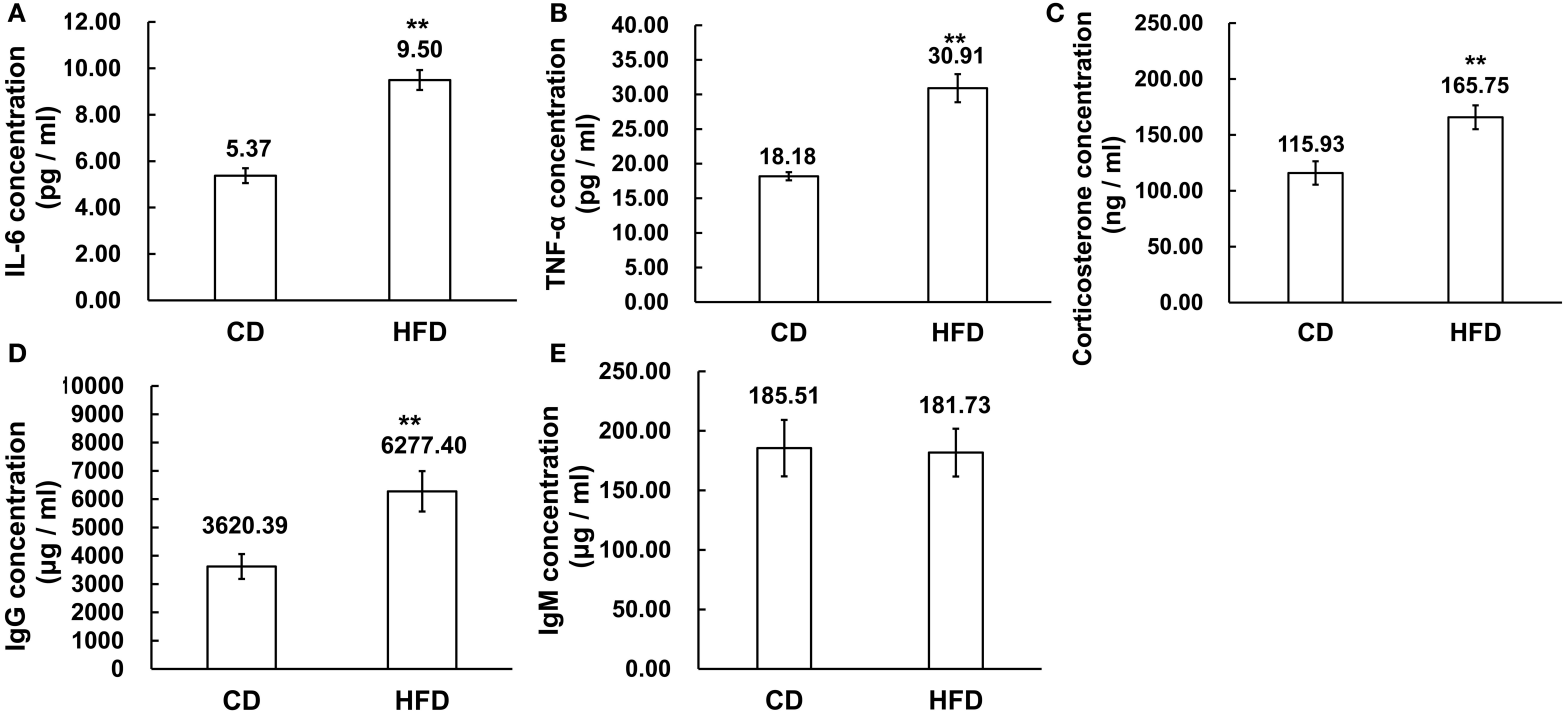
Alteration of inflammation related cytokines in sera. **(A,B)** Pro-inflammatory cytokines IL-6 and TNF-α expression levels were significantly higher in sera of mice fed a HFD than a CD. **(C)** Corticosterone concentration was higher in sera of mice fed a HFD than a CD. **(D,E)** Immunoglobulins, IgG, and IgM concentration, was significantly higher level of IgG in sera of mice fed a HFD than a CD while the IgM concentrations were unchanged in the CD and HFD mice sera ^**^P < 0.01.

The serous levels of IL-6, TNF-α, corticosterone, IgG, and IgM were measured by ELISA. The results demonstrated that obesity induced significantly higher expression levels of IL-6 and TNF-α in sera. In obese mice, IL-6 and TNF-α levels were obviously higher than those in the control group (IL-6: 9.50 ± 1.54 pg/ml vs. 5.37 ± 0.72 pg/ml, TNF-α: 30.91 ± 5.74 pg/ml vs. 18.18 ± 1.46 pg/ml, *n* = 10, *P* < 0.01, Figures 4A,B). On the other hand, the corticosterone level was significantly higher in obese mice than that in the control group (165.75 ± 37.01 ng/ml vs. 115.93 ± 37.74 ng/ml, *n* = 10, *P* < 0.01, Figure 4C). Meanwhile, the level of IgG was much higher in high-fat diet mice than that in control group (6227.40 ± 2256.22 μg/ml vs. 3620.39 ± 1390.46 μg/ml, *n* = 10, *P* < 0.01, Figure 4D), whereas the IgM concentration in serum was unchanged between high-fat diet and normal-diet mice (185.51 ± 78.65 μg/ml vs. 181.73 ± 80.31 μg/ml, *n* = 10, *P* > 0.05, Figure 4E).

### mRNA expression of pro-inflammatory cytokines and inflammasome in mice genital tract tissues

**Figure 5:**
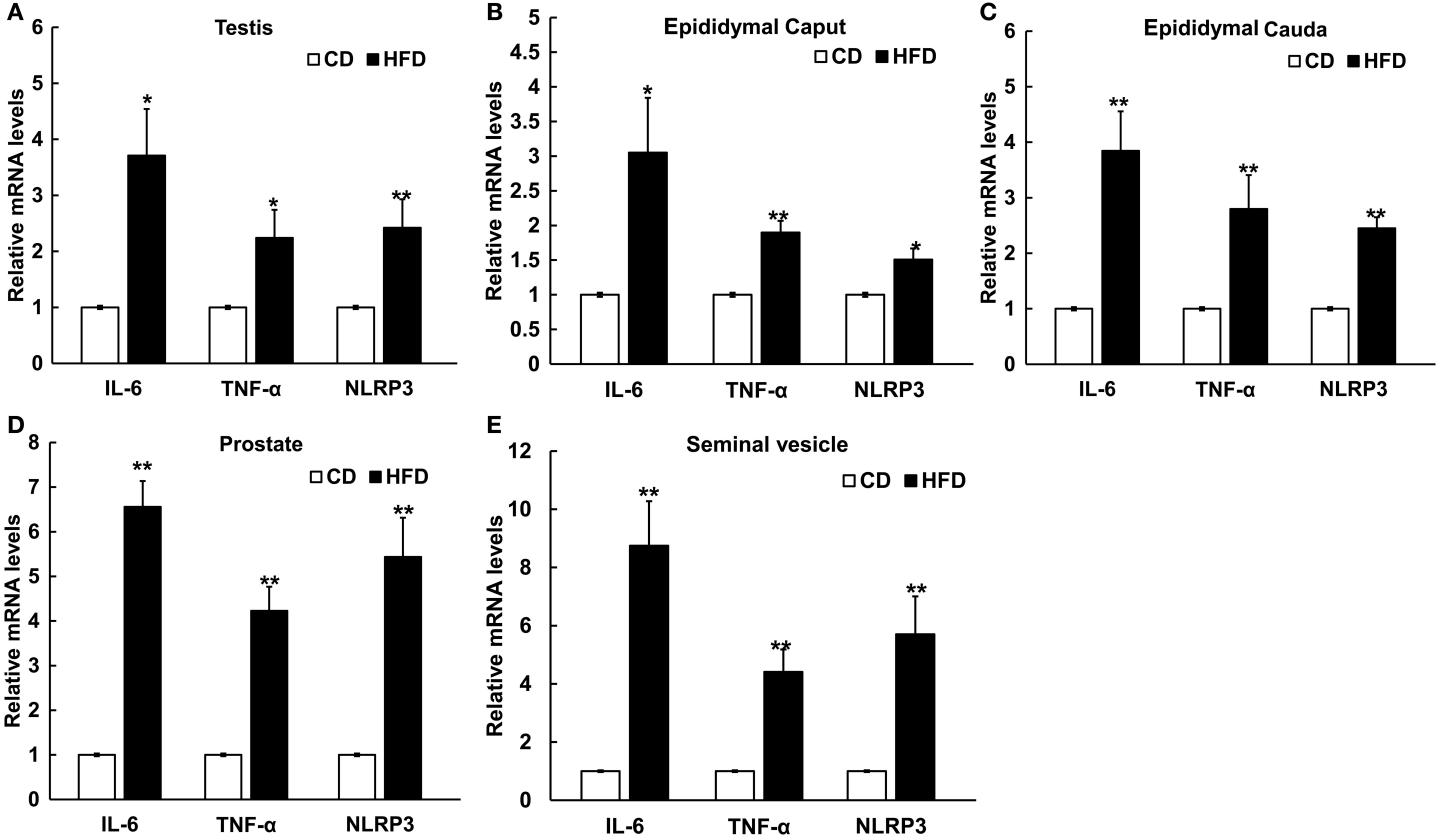
Differential expressions of pro-inflammatory cytokines and NLRP3 inflammasome protein in male genital tract. IL-6, TNF-α and NLRP3 mRNA levels in: **(A)** testis, **(B)** epididymal caput, **(C)** epididymal cauda, **(D)** prostate, and **(E)** seminal vesicle, and in all of these tissues mRNA levels significantly increased in HFD mice. ^*^P < 0.05, ^**^P < 0.01.

The mRNA levels of IL-6, TNF-α, and NLRP3 in testis, epididymal caput, epididymal cauda, prostate, and seminal vesicle tissues were measured to assess the inflammatory alteration in male genital tract from obese mice. The results of Real-Time PCR showed that the mRNA levels of IL-6, TNF-α, and NLRP3 remarkably increased in obese male genital tract. In testis, the mRNA expressions of IL-6, TNF-α, and NLRP3 were 3.7 ± 0.83, 2.24 ± 0.50, and 2.42 ± 0.51 folds higher in obese mice, respectively, than those in the normal group (*n* = 10, *P* < 0.05, Figure 5A). In the epididymal caput, the mRNA expression levels of IL-6, TNF-α, and NLRP3 were 3.05 ± 0.79, 1.90 ± 0.17, and 1.51 ± 0.16-folds higher, respectively, than those in the normal group (*n* = 10, *P* < 0.05, Figure 5B). In the epididymal cauda, the mRNA expression levels of IL-6 TNF-α, NLRP3 were 3.85 ± 0.71, 2.80 ± 0.61, and 2.45 ± 0.20-folds higher, respectively, than those in the normal group (*n* = 10, *P* < 0.05, Figure 5C). In the prostate, the mRNA expression levels of IL-6, TNF-α, and NLRP3 were about 6.56 ± 0.58, 4.23 ± 0.54, and 5.44 ± 0.88-folds higher, respectively, than those in the normal group (*n* = 10, *P* < 0.05, Figure 5D). Besides, in the seminal vesicle, the mRNA expression levels of IL-6, TNF-α, and NLRP3 were 8.75 ± 1.53, 4.41 ± 0.77, and 5.71 ± 1.30-folds higher in obese mice, respectively, than those in the normal group (*n* = 10, *P* < 0.05, Figure 5E). These results further validated that obesity can indeed induce a chronic inflammatory status in the male mice genital tract.

### Expression of p38, NF-κB, JNK, ERK, and their phosphorylated status, and NLRP3 in testis in high fat diet fed mice

**Figure 6:**
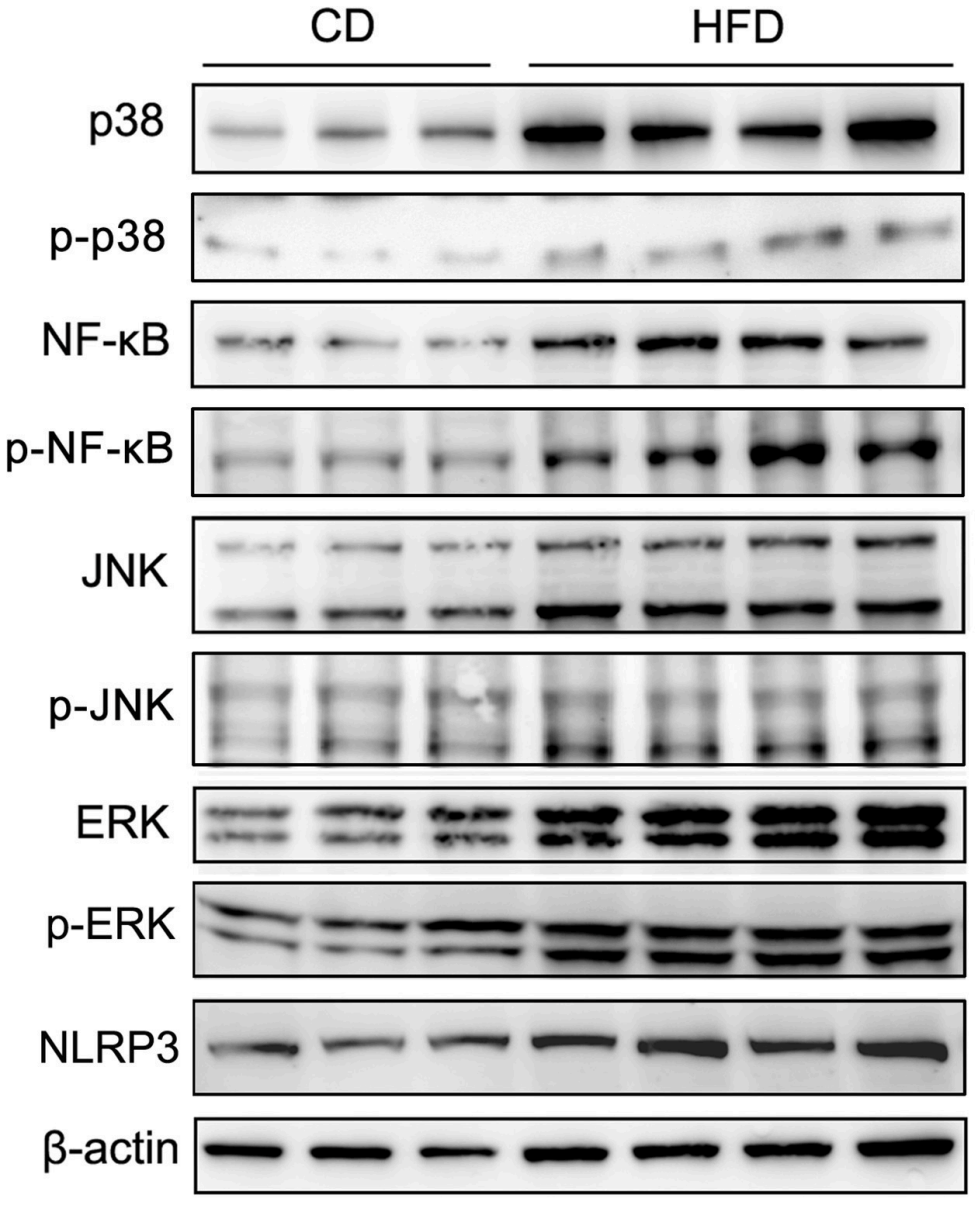
Alteration of testicular inflammation related protein levels. Western blot analyses of representative downstream signaling proteins mediating inflammation, include p38 MAPK, phosphorylated p38 (p-p38), NF-κB, phosphorylated NF-κB p65 (p-NF-κB), JNK, phosphorylated JNK (p-JNK), ERK1/2, phosphorylated ERK1/2 (p-ERK) and inflammasome protein NLRP3, showed that the levels of these inflammation related proteins in testes significantly increased in mice fed a HFD. β-actin expression invariance confirmed protein loading equivalence and normalized aforementioned expression levels.

To evaluate the effects of TNF-α expression rises on signaling pathway mediators in obese mice, we determined their effects on some key representatives. They included p38, NF-κB (p65), JNK, ERK1/2 (p44/42), phosphorylated p38 (p-p38), phosphorylated NF-κB p65 (p-NF-κB), phosphorylated JNK (p-JNK), phosphorylated ERK1/2 (p-ERK), and NLRP3 in testes. The results of Western blots showed that all of these effectors were upregulated in response to high-fat treatment (Figure 6).

### Expression levels of IL-6 and TNF-α in seminal plasma of normal, overweight, and obese human males

**Figure 7:**
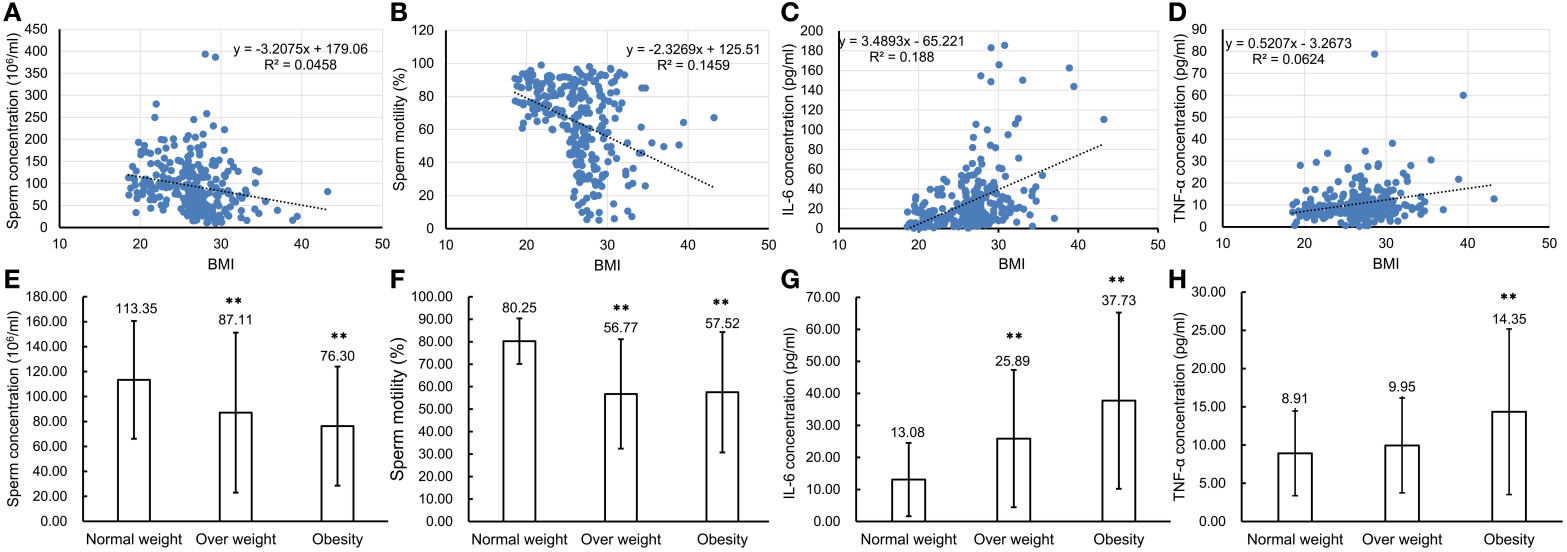
Comparison of parameter values used to assess sperm health and pro-inflammatory cytokine levels in seminal plasma of normal weight, overweight and obese males. **(A–D)** Correlations between BMI of the total 272 males and sperm concentration **(A)**, sperm motility **(B)**, IL-6 concentration **(C)** and TNF-α concentration **(D)**. Pearson r correlation calculations evaluated the association between BMI, sperm parameters and seminal plasma cytokine levels. **(E–H)** Comparison of sperm concentration **(E)**, sperm motility **(F)** with levels of IL-6 **(G)**, and TNF-α **(H)** in seminal plasma of normal weight, overweight and obese males. ^**^P < 0.01.

To confirm that chronic inflammation existed in the genital tract of overweight and obese males, 272 semen samples from normal weight, overweight and obese human males were collected and then analyzed accordingly. The CASA analyses of spermatozoa from both normal, overweight and obese males revealed that the sperm concentration and motility were inversely reverse correlated with BMI (*n* = 272, *P* < 0.01, Figures 7A,B). Especially, the sperm concentration and motility from overweight (sperm concentration: 87.11 ± 64.12 million sperm/ml and sperm motility: 56.77 ± 24.33%, *n* = 150, *P* < 0.01) and obese males (sperm concentration: 76.30 ± 47.73 million sperm/ml and sperm motility: 57.52 ± 26.78%, *n* = 40, *P* < 0.01) greatly decreased in comparison to those from the normal donors (sperm concentration: 113.35 ± 47.19 million sperm/ml and sperm motility: 80.25 ± 10.13%, *n* = 82, *P* < 0.01, Figures 7E,F). Simultaneously, the expression levels of IL-6 and TNF-α in seminal plasma measured by ELISA were also significantly correlated with BMI (*n* = 272, *P* < 0.01, Figures 7C, D). The results showed that IL-6 expression in the seminal plasma from overweight (25.89 ± 21.45 pg/ml, *n* = 150, *P* < 0.01) and obese males (37.73 ± 27.52 pg/ml, *n* = 40, *P* < 0.01) was obviously up-regulated in comparison to that from the normal males (13.08 ± 11.44 pg/ml, *n* = 82, Figure 7G). Meanwhile, TNF-α levels in seminal plasma from obese males (14.35 ± 10.83 pg/ml, *n* = 40, *P* < 0.01) was markedly elevated compared with that from normal males (8.91 ± 5.54 pg/ml, *n* = 82, Figure 7H).

## Discussion

Chronic inflammation is relevant to increases in adipose tissue content, which occurs along with the expression of abnormal adipocytokines, including several interleukins and tumor necrosis factor (Kaur, 2014). Moreover, high-fat diet feeding is known to induce increases in the secretion of intestinal pro-inflammatory cytokines and large increases in intestinal permeability (Li et al., 2008; Liu et al., 2012). In our previous findings, the male obese mouse model induced by high-fat diet can develop a remarkable impairment of sperm function, including reduced sperm motility, decreased acrosome reaction and fertility rate, and abnormal sperm morphology (Fan et al., 2015). Similar to male obesity, the pro-inflammatory state in males induced by chronic infection, smoking, and environmental toxins is also associated with subfertility phenotypes (Bachir and Jarvi, 2014). Thus, in the present study, our aim was to determine if there is a correlation between male obesity and chronic inflammation and elucidate its underlying mechanism.

Initially, an obese mouse model was successfully established by feeding a HFD which leads to development of an obese phenotype that includes significant increases in body weight, prominent rises in total cholesterol in, LDL, HDL, and apolipoprotein levels in the serum and serious hepatic steatosis. Besides, a remarkable endocrine dysregulation was detected in obese mice. Such defects were accompanied by very notable abnormal sex hormone expression patterns that included increased estradiol and decreased testosterone and progesterone levels.

Sex steroid hormones, such as estradiol, testosterone, and progesterone, regulate a considerable number of functions including reproduction, cell proliferation, and apoptosis as well as the responses to microbial or viral infections (Edwards, 2005). Sex steroid hormones can significantly modulate the activity of immune cells, i.e., as a protective factor, testosterone appears to suppress the activation and production of pro-inflammatory cytokines such as TNF-α, γ-interferon, and IL-6, in macrophages, lymphocytes, and vascular smooth muscle cells (McKay and Cidlowski, 1999; Malkin et al., 2003). On the other hand, estrogens can enhance both cellular and humoral immune responses and contribute to the resistance against infections through stimulating the synthesis of pro-inflammation cytokines including IL-6 and TNF-α (Vegeto et al., 1999; Straub, 2007). Moreover, it is well established that progesterone is an immunosuppressive agent that can blunt NF-κB activation (Su et al., 2009). Therefore, these previous reports concur with our present results, namely; the higher IL-6, TNF-α, and NF-κB levels detected in male genital tract tissues which may suppress testosterone and progesterone levels along with upregulating estradiol.

It is apparent that obesity can induce testicular inflammation through activating several different signaling pathways. In the male genital tract, testicular macrophages along with some proinflammatory cytokines such as IL-6 produced by Leydig and Sertoli cells contribute to the development of chronic inflammation (Maegawa et al., 2002). On the other hand, some immune regulatory factors can be secreted by Leydig and Sertoli cells in the testis. All of these factors contribute to regulating spermatogenesis and other testicular cell functions (Fraczek and Kurpisz, 2015). In addition, other proinflammatory cytokines as well as different immune regulatory factors are also produced in the epididymis and seminal vesicles (Huleihel and Lunenfeld, 2004; Seshadri et al., 2009). Actually, increases in the cytokines expression levels are indeed one of the first signals released by the innate host defense to counteract genital tract inflammation (Fraczek and Kurpisz, 2015). Consistent with this notion, our data shows that a prolonged high-fat diet could lead to increases in NLRP3 inflammasome and proinflammatory cytokine expression level such as IL-6 and TNF-α in the testis, epididymal caput, epididymal cauda, prostate, and seminal vesicle. Meanwhile, significant increases also occurred in IL-6 and TNF-α levels in sera. Besides, compared with the control group, corticosterone, and IgG concentrations were higher in obese mice sera. Notably, corticosterone, which acutely increases in response to inflammation through the hypothalamic–pituitary–adrenal (HPA) axis, has potent immunosuppressive and anti-inflammatory effects that are essential for regulating inflammation (Hueston and Deak, 2014). Such control is manifested by IgG which is the major antibody isotype in serum expressed by B lymphocytes and plasma cells. It provides immunity to inflammation, whereas IgM antibodies, have broad spectrum nonspecific effects because they recognize diverse microbial determinants and autoantigens. They are crucial for early protection against infection from viruses, bacteria, protozoa, fungi, and helminths, and control of autoimmunity. In addition, IgM exerts an important homeostatic function by clearing dead cells (Panda and Ding, 2015; Chistiakov et al., 2016; Pleass et al., 2016). Indeed we found that increases in corticosterone and IgG but not IgM in obese mice serum link obesity to chronic inflammation rather than infection or autoimmunity. Furthermore, our results clearly show that obesity can lead to systemic inflammation that includes a regional inflammatory reaction in the male genital tract, which is a crucial site for supporting sperm production, maturation, and sustaining its function.

Inflammation can harm the male genital tract by increasing reactive oxygen species (ROS) generation (Keck et al., 1998). In turn, excessive ROS levels must be continuously inactivated by seminal plasma antioxidants to maintain normal cell function. When free radicals become excessive, they overwhelm the genital tract antioxidant defense system. Such a scenario is indicative of an oxidative stress condition (Agarwal et al., 2003). Some previous reports indicated that seminal oxidative stress was negatively correlated with either sperm concentration, motility, or progressive motility (Kemal Duru et al., 2000). On the other hand, TNF-α levels were reported to increase in some tissues isolated from obese human populations and rodents showing such rises play a crucial role in obesity-induced inflammation (Tilg and Moschen, 2006). TNF-α can also inhibit tumorigenesis and viral replication through inducing apoptosis (Kruglov et al., 2008). Several studies have revealed that TNF-α is one of the major cytokines produced and released by macrophages and other mononuclear phagocytes. It plays an essential role in the development of inflammation and the activation of other molecules in signaling pathways. They include p38, NF-κB, JNK1/2, and ERK1/2 (Azenabor et al., 2015). In the testis, most of the germ cells undergo spontaneous degeneration during spermatogenesis and TNF-α determines the size of the germ cell population in the seminiferous epithelium by inducing germ cell apoptosis and disrupting Sertoli cell junctions as well as inhibiting steroidogenesis in Leydig cells (Lysiak, 2004). The high concentration TNF-α can alter intracellular Ca^2+^ homeostasis by decreasing plasma membrane permeability to Ca^2+^ (Carrasquel et al., 2013), whereas Ca^2+^ signaling mediated responses are one of the main factors regulating sperm function (Publicover et al., 2007). Many studies reported that Ca^2+^ signaling is involved in the regulation of dynein activity in mice sperm flagella (Lesich et al., 2012). Consequently, higher TNF-α levels are associated with reduced sperm motility and abnormal morphology (Perdichizzi et al., 2007; Pascarelli et al., 2016). Additionally, TNF-α is a NF-κB activator which is a transcription factor having multiple critical roles in the regulation of the immune responses. There is accumulating evidence to show that NF-κB can regulate male germ cell apoptosis. Meanwhile, apoptotic cell death is essential for limiting germ cell population expansion during spermatogenesis and its dysregulation may directly cause male infertility (Pentikäinen et al., 2002). In our experiments, we found that higher TNF-α and NF-κB expression was prominent in the testicular male genital tracts of mice on a high-fat diet. We also found that their spermatozoa function was impaired based on significant declines in motility, progressive, and acrosome reaction. Moreover, a remarkable lower pregnancy rate was evident in mated normal female mice (Fan et al., 2015).

Blood-testis barrier (BTB) is one of the most protective blood-tissue barriers in mammals. Meiosis I and II, spermiogenesis, and spermiation all occur in a specialized microenvironment behind these highly resistant shields (Cheng and Mruk, 2012). It is reported that the BTB function can be disrupted by cytokines such as TGF-β, IL-1, and TNF-α, but instead enhanced by testosterone (Lie et al., 2013). TNF-α upregulated FAS expression and triggered increases in apoptosis through the NF-κB pathway which ultimately disrupted the BTB in mouse Sertoli cells (Starace et al., 2005). These TNF-α effects agree with those induced by recombinant TNF-α which compromised the tight junctional integrity of cultured *in vitro* Sertoli cells (Siu et al., 2003). Besides, mitogen-activated protein kinase (MAPK) cascade activation is associated with testicular BTB disassembly (Wong and Cheng, 2005; Li et al., 2006). The MAPK cassette consists of the p38, ERK1/2, c-Jun N-terminal kinase (JNK), or ERK5 pathways that can undergo activation leading to testicular dysfunction in mice fed a HFD (Lie et al., 2013). In the present study, testicular morphological analysis revealed that the seminiferous epithelia were atrophied in obese mice. Furthermore, the continuity of cell adhesions between spermatogenic cells and Sertoli cells was severely compromised. All of these disruptive effects may be induced by excessive increases in proinflammatory cytokine expression levels. We showed that disruptive effects of increases in proinflammatory cytokines on the testicular microenvironment are attributable to increases in the expression levels and phosphorylation status of testicular p38, NF-κB, ERK, JNK, and NLRP3 as well as some inflammation related signaling proteins. Such effects suggest that they are potentially related to the male fertility declines stemming from perturbing hormonal biosynthesis, germ cell development as well as disrupting BTB integrity. Such effects agree with our previous study in which we showed that cell junction related proteins such as clathrin, ZO-1 and occludin expression was remarkably down-regulated in obese mice.

More important, in order to verify the correlation between being overweight or obese and sperm parameters as well as proinflammatory cytokine levels in semen plasma, semen samples were collected from 272 donors, including 82 normal weight, 150 overweight, and 40 obese individuals respectively. Based on the sperm parameters measured by CASA, we found a negative relationship between sperm concentration, motility, and BMI, which indicated that both overweight and obese males were associated with low sperm counts and declines in sperm motility. Moreover, the concentrations of IL-6 and TNF-α were measured in the semen plasma and the results clearly showed that their levels significantly increased in the semen plasma from obese or overweight males compared with that of normal body weight individuals. These observations further demonstrated that obesity or overweight can indeed up-regulate cytokine concentrations in the male genital tract and impair sperm quality. These changes are consistent with those occurring in mice on a HFD. These results confirm previous data showing that there is a negative correlation between cytokine levels in the semen plasma and semen quality based on declines in sperm concentration (Furuya et al., 2003; Sanocka et al., 2003), motility, and progressive motility (Matalliotakis et al., 2006).

In conclusion, this study demonstrates that excessive body weight and obesity in humans is associated with development of an inflammatory status in the male genital tract. This condition constitutes one of the risk factors leading to male infertility. It stems from increases in proinflammatory cytokine levels that can impair male fertility via inducing germ cell apoptosis and compromising testicular BTB integrity. Such effects eventually adversely impair the biological functions of mature gametes. Furthermore, in parallel with this study, our clinical experiments also indicate that in comparison to males who have normal body weight, the obese or overweight individuals had poor semen quality combined with higher levels of IL-6 and TNF-α in the semen plasma. This close correspondence between the clinical signs and functional changes seen in obese mice on a HFD with those in overweight and obese human males suggest that reducing chronic testicular inflammation provides a novel therapeutic option to reduce male infertility in humans.

## Author contributions

WF, YX, and YL collected and prepared clinical samples and data, and conducted experiments, data generation and analysis, and manuscript preparation. ZZ performed experiments and collected data. LL and ZD was responsible for the conception and design, supervision of all aspects of the laboratory experiments, data analysis and the preparation, and final approval of the manuscript.

### Conflict of interest statement

The authors declare that the research was conducted in the absence of any commercial or financial relationships that could be construed as a potential conflict of interest.
